# Hemp Pest Spectrum and Potential Relationship between *Helicoverpa zea* Infestation and Hemp Production in the United States in the Face of Climate Change

**DOI:** 10.3390/insects12100940

**Published:** 2021-10-15

**Authors:** Olufemi S. Ajayi, Michelle Samuel-Foo

**Affiliations:** Department of Biological Sciences, Alabama State University, Montgomery, AL 36104, USA; mfoo@alasu.edu

**Keywords:** industrial hemp, *Cannabis sativa*, climate change, pests, beneficials, corn earworm

## Abstract

**Simple Summary:**

Cultivation of industrial hemp *Cannabis sativa* in the United States is now being expanded due to the recent legalization of the crop. Multiple insect pests attack the crop. One of the common pests is the corn earworm *Helicoverpa zea* that causes extensive damage to the marketable parts of hemp. Changing global climate may lead to expansion of the geographic range of insect pests. Thus, growers of this crop in the United States have to face new and intense pest problems now and in the years to come. Here, we assess the potential relationship between corn earworm infestation and hemp production in the US in the face of climate change. We also provide an update on the arthropods associated with hemp cultivation across the US. Climate change can affect aspects of interactions between hemp and corn earworm. Temperature and photoperiod affect the development and diapause process in *H. zea*. Drought leads to a reduction in hemp growth. Overall, our assessment suggests the selection of varieties resistant to stresses from climate and insects. Host plant diversity may prevent populations of corn earworm from reaching outbreak levels. Ongoing research on effective management of *H. zea* on hemp is critical.

**Abstract:**

There has been a resurgence in the cultivation of industrial hemp, *Cannabis sativa* L., in the United States since its recent legalization. This may facilitate increased populations of arthropods associated with the plant. Hemp pests target highly marketable parts of the plant, such as flowers, stalks, and leaves, which ultimately results in a decline in the quality. Industrial hemp can be used for several purposes including production of fiber, grain, and cannabidiol. Thus, proper management of pests is essential to achieve a substantial yield of hemp in the face of climate change. In this review, we provide updates on various arthropods associated with industrial hemp in the United States and examine the potential impact of climate change on corn earworm (CEW) *Helicoverpa zea* Boddie, a major hemp pest. For example, temperature and photoperiod affect the development and diapause process in CEW. Additionally, drought can lead to a reduction in hemp growth. Host plant diversity of CEW may prevent populations of the pest from reaching outbreak levels. It is suggested that hemp varieties resistant to drought, high soil salinity, cold, heat, humidity, and common pests and diseases should be selected. Ongoing research on effective management of CEW in hemp is critical.

## 1. Introduction

**Figure 1:**
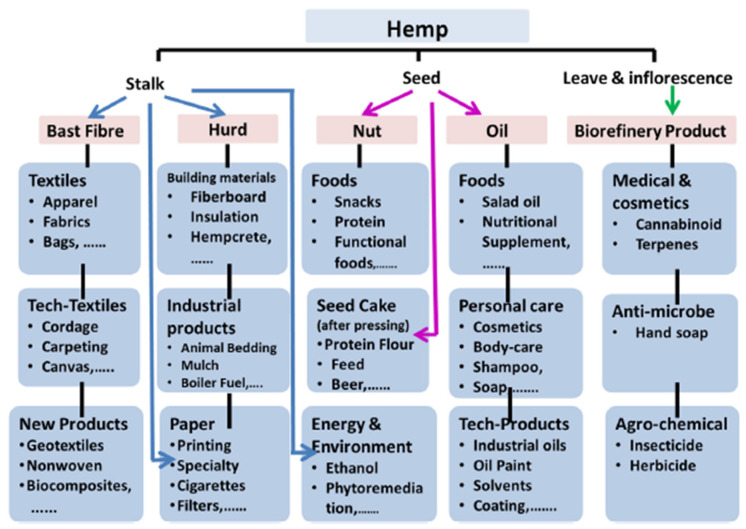
Flowchart of multi-purpose hemp utilization. Graphic is from Salentijn et al. [4].

Industrial hemp or hemp (*Cannabis sativa* L.) cultivation is assuming new geographical borders around the world [1,2,3]. It is of medicinal, industrial, and economic importance. It is usually cultivated for production of long and strong bast fibers, seeds, oil, and food (Figure 1) [4]. Hemp contains extremely low amounts of the psychoactive cannabinoid Δ 9-tetrahydrocannabinol (THC). Cannabis plants that contain a concentration of less than 0.3% THC are considered hemp; while those above this concentration are considered marijuana [5]. Here, we regard industrial hemp as hemp varieties with amounts of less than 0.3% THC.

**Figure 2:**
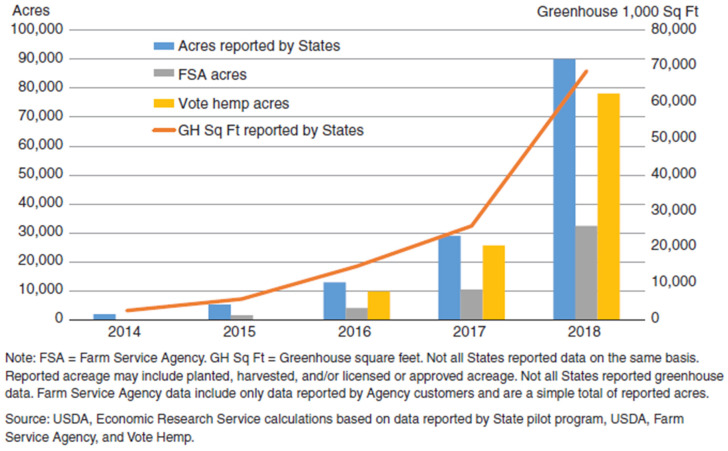
United States hemp acreage and greenhouse area, reported 2014–2018. Graphic is from Tyler et al. [7].

The legality of hemp cultivation varies worldwide. A unique aspect of hemp history in the US is the ban of its cultivation in 1937 when the federal Marihuana Tax Act effectively criminalized almost all cannabis cultivation [6]. The Agricultural Act of 2014 (also called the 2014 Farm Bill) reintroduced industrial hemp production through state pilot programs [7]. In 2018, the Agriculture Improvement Act, also known as the 2018 Farm Bill, re-legalized commercial hemp production in the US [8]. These decades of prohibition of cultivation resulted in little to no research on hemp in the US. Since 2014, however, legalization of industrial hemp in the US has resulted in increased interest in the cultivation of the crop (Figure 2) [7,9]. Despite this increase, the hemp industry is still regarded as emerging; and there is a lack of established production methods around the country [10]. This has resulted in many producers modifying and experimenting with hemp production. Presently, there are efforts around North America to develop improved cultivars for production of one or more of the commodities derived from industrial hemp [4,11,12]. Some varieties are being developed for improvement of their CBD contents, fiber production, and grain content [7]. Stakeholders in the US want breeding and genetics research to produce stable and uniform cultivars and regional adaptability [13].

Growing conditions for hemp cultivation are documented in the literature [14]; however, optimal growing conditions are expected to vary according to cultivar. An important aspect of hemp cultivation is the management of arthropod pests. As with any crop, successful cultivation of hemp can include integrated pest management strategies. Efficient management of arthropod pests on hemp starts with surveying and properly identifying its insect community. There are reports of arthropods associated with hemp globally [15] and within the US [12]. However, with the current expansion in the cultivation of hemp across several states, reports/knowledge of arthropod pests needs to be updated. Furthermore, surveys have suggested negative grower experiences with hemp production especially from first-time or inexperienced hemp growers [10]. This highlights the need to educate growers on arthropod communities and pest control on hemp.

One of the most important challenges facing agriculture worldwide is management of abiotic stressors, including increasing temperatures and prolonged periods of drought. Such climatic anomalies are expected to drive the spread of arthropods [16,17], including those associated with hemp. In addition, biotic factors play a decisive role in species spatial distributions presently and will continue under future climate change [18]. The market value of industrial hemp in the US is impacted due to several pest insect species increasingly located on the crop [12]. Some authors have previously reviewed arthropods of hemp. Mostafa and Messenger [19] reported about 272 species of insect and mite species associated with *Cannabis* globally. McPartland et al. [15] described about 150 species of insects and mites associated with hemp. Cranshaw et al. [12] described several arthropod pests associated with the production of hemp and the associated pest management needs in the US. Here, we review and update the arthropods (both pests and beneficials) affecting industrial hemp across the US. Furthermore, we discuss how climate change could affect one of the prevalent pests.

## 2. Industrial Hemp Pests

Many phytophagous insects feed on industrial hemp, though only some species attain pest status [12,15]. Cranshaw et al. [12] arranged arthropods on hemp across the United States into the following categories—pests: defoliators, sucking insects and mites on leaves, stem and stalk borers, sucking insects associated with flowers and seeds, chewing insects that damage flower buds and seeds, and root feeders; natural enemy species: predators, parasitoids, pathogens; and pollinators.

Some pests and beneficial arthropods reported on hemp in the US are listed in Table 1 and Table 2, respectively. Some arthropods that are currently considered as neither pest nor beneficial to hemp that exist in the US are listed in Table 3.

There are some challenges to sampling in hemp, therefore future directions should include some standardization of methods. For example, there are limitations in using just a visual count in comparison to sweep-net and beat-into-alcohol methods. Other methods of collection used are pitfall traps and yellow sticky cards. A factor that could strongly influence sampling in hemp is that neighboring crops to hemp can impact insect community. Furthermore, weather pattern can impact the insect types and the population densities.

## 3. Corn Earworm and Hemp: Potential Effects of Climate Change

Corn earworm *Helicoverpa zea* (Boddie, 1850) (Lepidoptera: Noctuidae) is native to the Americas [15]. *Helicoverpa zea* is very common on hemp plants across the United States (Table 1). It is a polyphagous, multivoltine insect pest and has a wide range of hosts, including many vegetables, field crops, fruits, flowers, and weeds. It causes serious damage in several crops, including corn, tomato, pepper, cotton, sorghum, and lettuce [34]. Around the world, it is called by a plethora of common names, including corn earworm, cotton bollworm, tobacco budworm, tobacco fruitworm, and vetchworm [15].

*Helicoverpa zea* overwinters as a pupa within the soil; in the US, successful overwintering occurs only in the southern USA, as *H. zea* cannot successfully overwinter at areas above 39° N [15,35]. Adults emerge from overwintering pupae in spring, and can then migrate throughout most of the United States and southern Canada during the growing season [36]. Adults mate and females lay eggs in floral inflorescences. A single female can lay up to 1500 eggs in her lifetime [37,38]. Emerged larvae feed on and injure hemp bud material, causing “bud rot” [39]. In North America, *H. zea* produces one to seven generations per year, depending on the latitude (e.g., one to two generations in Ontario, seven generations in south Texas) [40,41,42,43,44]. Like most multivoltine insects, its development and diapause termination are expected to be driven by temperature, while its diapause initiation triggered by photoperiod [45]. *Helicoverpa zea* pupae are chill-intolerant, thus they are unable to withstand freezing and are subject to much prefreeze mortality [46]. Sex does not influence the cold response of *H. zea* pupae [46,47]. Enhanced cold hardiness is gained through diapause in *H. zea* pupae [46,47,48]. It has been predicted that *H. zea* would respond to climate change by altering its voltinism [49].

Over the last decades, many studies on climate trends have been carried out and results demonstrate that patterns of temperature and precipitation are rapidly shifting, affecting large parts of our planet, in both animals and plants [50,51,52,53,54]. Some plant and animal species may react to climate change by showing some degree of adaptation and mitigation of its effects [55,56]. It is expected that over the coming decades, many plant and animal species will be affected in all aspects of their biology [57], and that adaptation to counterbalance impacts of climate change will be a challenge. Several aspects of insect biology can be affected by warmer temperatures, including survival and reproduction [58]. Furthermore, climate change can impact aspects of plant-insect interactions including host resistance and quality [59,60,61,62]. Natural enemies of pest insects can also be impacted by climate change [63,64,65]. These natural enemies, including parasitoids and predators, are dependent on the resilience of their host insects in the face of climate change, thus further exacerbating the stress on them [64]. 

In the US, climate change is a growing threat to biodiversity and ecosystems, and their services [66]. For example, in the Mid-Atlantic region of the US, where *Cannabis* hemp is widely cultivated, it is anticipated that by mid- to late- century (i.e., 2035–2049 and 2085–2099), there will be a warmer climate with a wetter Autumn and Spring and a drier late summer season; this is expected to cause damage to plants [67]. An increasing number of studies show a link between drought and reduced hemp growth, including stem and fiber yield e.g., [68]. Furthermore, climate change is making the western US more arid [69]. This has contributed to drier soils [70], widespread plant death [71], and more severe wildfires [72]. Hemp is a tall plant with a wide root system (at least 0.5 m deep) and it is a good candidate for soil phytoremediation as it grows fast and easily in dense stands [73]. However, several environmental conditions such as drought, flooding, heat, and salinity affect the level of hormones in plants [68,74,75,76,77,78]. For example, under water stress, there is reduced transport of cytokinins from the root (the site of biosynthesis) to shoots [79,80]. This reduction in cytokinin is expected to bring about a shift towards maleness [78,81]. If this occurs in *Cannabis* plants, then it would ultimately influence the quantity and type of insects such as pollinators on hemp plants. Furthermore, it has been predicted that the US may experience warmer winters, resulting in diminished vernalization [82,83], a process required to promote flowering in certain types of crops. It is suggested that hemp varieties resistant to drought, high soil salinity, cold, heat, humidity, and common pests and diseases should be selected [84].

The mouthpart type of phytophagous insects influences their reaction to stress-induced plant changes [85]. For instance, decreased water content, tougher foliage, elevated levels of allelochemicals and reduced nitrogen availability all reduce nutritional quality of host plant tissue for chewing insects (e.g., corn earworm) [86]. Corn earworm herbivory increases the levels of chemical defense in cotton and has caused a significant decline in the nutritional quality of the plant as a host [87]. A similar severe decline in the nutritional quality of other plants such as soybean, geranium, and clover also occurred by corn earworm herbivory [88,89,90,91]. Recent studies have demonstrated that infestations of corn earworm on *C. sativa* increases the levels of cannabidiol (CBD) and delta-9-tetrahydrocannabinol (THC) beyond the 0.3% legal limit [92].

Some of the most prevalent and consistent pests of hemp are also major insect pests of corn (*Zea mays* L.). These include the European corn borer [*Ostrinia nubilalis* (Hübner)] and corn earworm [*Helicoverpa zea* (Boddie)]. Studies have shown that *O. nubilalis* antennae consistently responded to at least four chemical compounds, which all co-occur in both corn and hemp [93]. This suggests that these plant volatile compounds are cues used by these insects in herbivory. More research into the role of plant volatile compounds in the mechanisms of host location by these pests on hemp plants is needed.

Though outbreaks of *H. zea* have occured in some regions of the United States where *C. sativa* is cultivated, host-plant diversity may prevent populations of *H. zea* from reaching outbreak levels [94]. Host plants of the generalist *H. zea* larvae include corn, tomato, and cotton, which are all economically important crops in the United States. *H. zea* is regarded as a serious pest of these crops making effective management of *H. zea* necessary. In the evaluation of biological insecticides to manage *H. zea* in CBD hemp in Virginia, Entrust SC (Spinosad) had a significantly lower incidence of bud rot than all other treatments [95]. Furthermore, no signs of phytotoxicity were observed from any of the biopesticide treatments in the study. In a similar study, Entrust (Spinosad) resulted in significantly fewer corn earworms and less damage than untreated control [96]. Furthermore, in another study, Entrust (Spinosad) resulted in a significantly higher corn earworm mortality (95%) than any other tested biological or organic insecticide products on field-collected corn earworms tested in laboratory assays after four days [97]. In a second bioassay, Pyganic and Entrust performed significantly better than all other treatments, resulting in 100% and 97.5% respective mortality in lab-reared corn earworms [97]. A likely reason for the difference between lab-reared and wild-caught populations could be that resistance to Cry1AB Bt proteins is widespread in Virginia corn earworms [97].

Stressed plants are expected to have reduced defenses and therefore greater vulnerability to herbivores (plant stress hypothesis; [98,99,100]). A similar pattern might be expected with the vulnerability of stressed *C. sativa* plants to herbivores such as *H. zea*. However, the plant vigor hypothesis contradicts this. For example, Inbar et al. [101] reported that larval growth rates of *H. zea* were higher on tomato (*Lycopersicon esculentum*) exposed to optimal growing conditions, but lower on those exposed to stress. Whether stressed or not, morphological defense mechanisms of *C. sativa* may override the extent to which chewing herbivores such as *H. zea* damage the plant. For example, *H. zea* was negatively affected by trichome density on yellow monkey flower *Mimulus guttatus* [100]. This may be similar to the response of *H. zea* to stressed *C. sativa*.

## 4. Conclusions

*Helicoverpa zea* is a polyphagous insect pest on hemp, *Cannabis sativa*. In North America, the pest produces one to seven generations per year, depending on the latitude. Like most multivoltine insects, its development and diapause termination are expected to be driven by temperature, while its diapause initiation by photoperiod. *Helicoverpa zea* pupae are chill-intolerant, thus subject to much prefreeze mortality. *H. zea* could respond to climate change by altering its voltinism. Furthermore, climate change can affect aspects of interactions between hemp and corn earworm, including host resistance and quality. Natural enemies of corn earworm are dependent on the resilience of their host in the face of climate change. Water stress on hemp could bring about a shift towards maleness in the plant, and this could ultimately influence the quantity and type of insects such as pollinators on hemp. Drought leads to a reduction in hemp growth. Infestations of corn earworm on hemp increases the level of THC beyond the 0.3% threshold point at which cannabinoid content is used to distinguish strains of hemp from marijuana. Plant volatile compounds could be involved in cues used by *H. zea* in herbivory. Though outbreaks of corn earworm have been experienced in some regions of the United States where hemp is cultivated, host-plant diversity may prevent populations of corn earworm from reaching outbreak levels in regions with such diversity compared with those without. Ongoing research on effective management of *H. zea* on hemp is critical. Future research should focus on understanding abiotic stress responses in hemp, corn earworm and its natural enemies in hemp. Impacts of climate change on industrial hemp production mediated through changes in populations of serious insect pests such as corn earworm need to be given more attention for planning and devising adaptation and mitigation strategies for future management programs. There are still gaps in information that need to be addressed in order to allow production of management plans for these arthropod pests. It is also necessary to encourage the conservation of beneficial insects on hemp, to use these arthropods as pest control strategies.

## Figures and Tables

**Table 1:** A list of some pest arthropods reported on hemp in the United States.

Family	Common Name	Scientific Name (for Those Identified to Species)	Damage Type	Location Found	References
Acrididae	Grasshopper		Pest	Field	[20]
Aeolothripidae	Thrips		Pest	Field	[20,21]
Aphididae	Cannabis aphid	*Phorodon cannabis*		Field & greenhouse	[20,21,22,23,24,25]
Cercopidae	Spittlebug		Pest	Field	[20]
Chrysomelidae	e.g., Spotted cucumber beetle, Leaf beetle	*Diabrotica undecimpunctata*; *Diabrotica v. virgifera*;	Herbaceous pest	Field	[20,21,26]
Cicadellidae	Leafhoppers, e.g., Beet leafhopper	e.g., *Circulifer tenellus*	Pest (some transmits beet curly top virus)	Field	[20,21,27,28]
Coreidae	Leaf-footed bug		Sucking-piercing pest	Field	[26]
Crambidae	European corn borer	*Ostrinia nubilalis*	Pest	Field	[21]
Curculionidae	Weevil		Herbaceous pest	Field	[20,26]
Elateridae	Click beetle		Pest	Field	[20]
Formicidae	Fire ant	*Solenopsis invicta*	Pest	Field	[20,23]
Meloidae	Blister beetle		Herbaceous pest	Field	[26]
Membracidae	Treehopper		Pest	Field	[20]
Miridae	Tarnished plant bug	*Lygus lineolaris*	Sucking-piercing pest	Field	[20,26]
Noctuidae	Corn earworm	*Helicoverpa zea*	Primarily, laceration of reproductive branch tip	Field	[23,26,28,29,30,31,32]
Pentatomidae	Stink bug		Sucking-piercing pest	Field	[20,26,28]
Rhopalidae	Hibiscus scentless plant bug	*Niesthrea louisianica*	Sucking-piercing pest	Field	[26]
Rhyparochromidae	Seed bug		Pest	Field	[20]
Scarabaeidae	Scarabs, e.g., Japanese beetle, Green June beetle	e.g., *Popillia japonica*	Herbaceous pest	Field	[20,21,26]
Tarsonemidae	Broad mites	*Polyphagotarsonemus latus*	Pest	Greenhouse	[28]
Tetranychidae	Two-spotted spider mite	*Tetranychus urticae*	Pest	Greenhouse	[29,31]
Tortricidae	Euroasian hemp borer (adults & larvae)	*Grapholita delineana*	Pest	Field	[20,23]

**Table 2:** A list of some beneficial arthropods reported on hemp in the United States.

Family	Common Name (If Any)	Scientific Name (for Those Identified to Species)	Association Type	Location Found	References
Anthocoridae	Insidious flower bug	*Orius insidiosus*	Beneficial	Field	[20]
Anthicidae	Ant-like beetle		Beneficial	Field	[20]
Araneae	Spiders		Natural enemy (predator)	Field	[20,33]
Braconidae	Braconids	*Cardiochiles* spp.	Natural enemy (parasitoid)	Field	[33]
Carabidae	Tiger beetles		Beneficial	Field	[20]
Chrysopidae	Green lacewing		Natural enemy (predator)	Field	[20,26]
Coccinellidae	Lady beetle	*Hippodamia convergens*; *Coleomegilla maculata*; *Hyperaspis lugubris*; *Cycloneda munda*; *Cycloneda sanguinea*; *Harmonia axyridis*	Natural enemy (predator)	Field & greenhouse	[20,24,26]
Dolichopodidae	Long-legged flies		Beneficial	Field	[20]
Geocoridae	Big-eyed bug	*Geocoris* spp.	Natural enemy	Field	[26]
Hemerobiidae	Brown lacewings		Beneficial	Field	[20]
Ichneumonidae	Ichneumonids		Natural enemy (parasitoid)	Field	[33]
Nabidae	Damsel bugs		Beneficial	Field	[20]
Pentatomidae	Spined soldier bug	*Podisus maculiventris*	Natural enemy	Field	[26]
Reduviidae	Assassin bug		Beneficial	Field	[20]
Syrphidae	Syrphid larvae		Natural enemy (predator)	Field	[20,33]
Tachinidae	Tachinids		Natural enemy (parasitoid)	Field	[33]
Vespidae	Paper wasps		Natural enemy (predator)	Field	[33]
	Opiliones (spider)		Beneficial	Field	[20]

**Table 3:** A list of some arthropods considered neither pest nor beneficial reported on hemp in the United States.

Family	Common Name (If Any)	Association Type	Location Found	References
Cerambycidae	Longhorn beetle	Other	Field	[20]
Cleridae	Checkered beetles	Other	Field	[20]
Gryllidae	Cricket	Other	Field	[20]
Latridiidae	Minute brown scavenger beetles or fungus beetle	Other	Field	[20]
Mordellidae	Tumbling flower beetles	Other	Field	[20]
Nitidulidae	Sap beetle	Other	Field	[20]
Pieridae	Pierid butterfly	Other	Field	[20]
Silvanidae	Silvan flat bark beetles	Other	Field	[20]
Staphylinidae	Rove beetle	Other	Field	[20]
Tipulidae	Crane fly	Other	Field	[20]
	Caddisflies (in the order Trichoptera)	Other	Field	[20]
	Centipede (in the class Chilopoda)	Other	Field	[20]
	Millipede (in the class Diplopoda)	Other	Field	[20]
	Booklice, barklice or barkflies (in the order Psocoptera)	Other	Field	[20]
	Leaf mining fly (larvae)	Other	Field	[28]

## Data Availability

Not applicable.

## References

[1] Shahzad A. (2012). Hemp fiber and its composites—A review. J. Compos. Mater..

[2] Tang K., Struik P.C., Yin X., Thouminot C., Bjelková M., Stramkale V., Amaducci S. (2016). Comparing hemp (*Cannabis sativa* L.) cultivars for dual-purpose production under contrasting environments. Ind. Crop. Prod..

[3] Fortenbery T.R., Bennett M. (2004). Opportunities for commercial hemp production. Rev. Agr. Econ..

[4] Salentijn E.M.J., Zhang Q., Amaducci S., Yang M., Trindade L.M. (2015). New developments in fiber hemp (*Cannabis sativa* L.) breeding. Ind. Crop. Prod..

[5] Small E., Cronquist A. (1976). A practical and natural taxonomy for *Cannabis*. Taxon.

[6] Johnson N. (2019). American Weed: A History of Cannabis Cultivation in the United States. EchoGéo.

[7] Tyler M., Shepherd J., Olson D., Snell W., Proper S., Thornsbury S. (2020). Economic Viability of Industrial Hemp in the United States: A Review of State Pilot Programs.

[8] (2018). Agriculture Improvement Act of 2018: Public Law Number 115-334, United States of America. https://www.govinfo.gov/app/details/PLAW-115publ334.

[9] Witkowski T.H. Cannabis marketing systems and social change in the United States. Proceedings of the 40th Annual Macromarketing Conference.

[10] Cui X., Smith S.A. (2000). University of Tennessee Extension’s 2020 Hemp Industry Survey. www.utia.tennessee.edu.

[11] Fike J. (2016). Industrial hemp: Renewed opportunities for an ancient crop. Crit. Rev. Plant Sci..

[12] Cranshaw W., Schreiner M., Britt K., Kuhar T.P., McPartland J., Grant J. (2019). Developing Insect Pest Management Systems for Hemp in the United States: A Work in Progress. J. Integr. Pest Manag..

[13] Ellison S. (2021). Hemp (*Cannabis sativa* L.) research priorities: Opinions from United States hemp stakeholders. Glob. Change Biol. Bioenergy.

[14] Adesina I., Bhowmik A., Sharma H., Shahbazi A. (2020). A review on the current state of knowledge of growing conditions, agronomic soil health practices and utilities of hemp in the United States. Agriculture.

[15] McPartland J.M., Clarke R.C., Watson D.P. (2000). Hemp Diseases and Pests: Management and Biological Control.

[16] Ladányi M., Horváth L. (2010). A review of the potential climate change impact on insect populations–General and agricultural aspects. Appl. Ecol. Environ. Res..

[17] Ajayi O.S., Appel A.G., Chen L., Fadamiro H.Y. (2020). Comparative cutaneous water loss and desiccation tolerance of four *Solenopsis* spp. (Hymenoptera: Formicidae) in the Southeastern United States. Insects.

[18] Leach K., Montgomery W.I., Reid N. (2016). Modelling the influence of biotic factors on species distribution patterns. Ecol. Model..

[19] Mostafa A.R., Messenger P.S. (1972). Insects and mites associated with plants of the genera, Argemone, Cannabis, Glaucium, Erythroxylum, Eschscholtzia, Humulus and Papaver.

[20] Anderson R., Zaric M., Rilakovic A., Kruger G., Peterson J. Diversity and Abundance of Arthropods in Industrial Hemp Fields of Nebraska. Proceedings of the Entomological Society of America Annual Meeting.

[21] Lewins S., Darby H. Insect pests of industrial hemp in the Northeastern US. Proceedings of the Entomological Society of America Annual Meeting.

[22] Villa E., Pitt W.J., Nachappa P. Cannabis aphid (Hemiptera: Aphididae), a New Vector of Potato Virus Y Infecting Hemp. Proceedings of the Entomological Society of America Annual Meeting.

[23] Nixon J., Samuel-Foo M., Kesheimer K. Insect Pests of Industrial Hemp. Proceedings of the Entomological Society of America Annual Meeting.

[24] Villanueva R.T., Viloria Z.J., Klueppel R., Bradley C. Biology, Damage, Suitability as Prey of Ladybugs, and Chemical Control of the Cannabis Aphid. Proceedings of the Entomological Society of America Annual Meeting.

[25] Lemay J., Scott-Dupree C. Biological Control in Odour Space: The Next Frontier. Proceedings of the Entomological Society of America Annual Meeting.

[26] Zobel E., Fiorellino N.M., Ristvey A. An Overview of the Insect Community Found in Fiber and Grain Industrial Hemp Grown on the Eastern Shore of Maryland in 2020. Proceedings of the Entomological Society of America Annual Meeting.

[27] Chiginsky J., Langemeier K., White S., Cranshaw W.S., Fulladolsa A.C., Stenglein M., Nachappa P. Hemp Virome Revealed, and the Ecology of Beet Curly Top Virus in Hemp in Colorado. Proceedings of the Entomological Society of America Annual Meeting.

[28] Burrack H.J., Ganji N., Pulkoski M. Arthropod Pest Management of Hemp in North Carolina. Proceedings of the Entomological Society of America Annual Meeting.

[29] Pulkoski M., Burrack H.J. Method Development for Plant Response to Insect Feeding Modes Study in Industrial Hemp (*Cannabis sativa*). Proceedings of the Entomological Society of America Annual Meeting.

[30] Cosner J., Grant J.F., Kelly H. Influence of Hemp Variety and Fertilizer Rate on Populations of Corn Earworm, *Helicoverpa zea*, and Plant Characteristics. Proceedings of the Entomological Society of America Annual Meeting.

[31] Fritz B. Progress in Biopesticides for Hemp IPM. Proceedings of the Entomological Society of America Annual Meeting.

[32] Zebelo S., Jackson B., Gilbert L., Tolosa T., Volkis V. Impact of Insect Herbivores on the Δ9THC and CBD Levels in Hemp. Proceedings of the Entomological Society of America Annual Meeting.

[33] Grant J., Hale F. Beneficials on Hemp: What You Need to Know. Proceedings of the Entomological Society of America Annual Meeting.

[34] Cranshaw W., Shetlar D. (2018). Garden Insects of North America: The Ultimate Guide to Backyard Bugs.

[35] Hardwick D.F. (1965). The corn earworm complex. Mem. Entomol. Soc. Can..

[36] Reay-Jones F.P.F. (2019). Pest status and management of corn earworm (Lepidoptera: Noctuidae) in field corn in the United States. J. Integr. Pest Manag..

[37] Akkawi M.M., Scott D.R. (1984). The effect of age of parents on the progeny of diapaused and non-diapaused *Heliothis zea*. Entomol. Exp. Appl..

[38] Fitt G.P. (1989). The ecology of *Heliothis* species in relation to agroecosystems. Annu. Rev. Entomol..

[39] Britt K.E., Kuhar T.P., Cranshaw W., McCullough C.T., Taylor S.V., Arends B.R., Burrack H., Pulkoski M., Owens D., Tolosa T.A. (2021). Pest management needs and limitations for corn earworm (Lepidoptera: Noctuidae), an emergent key pest of hemp in the United States. J. Integr. Pest Manag..

[40] Quaintance A.L., Brues C.T. (1905). The cotton bollworm. USDA Bur. Ent. Bul..

[41] Brazzel J.R., Newsom L.D., Roussel J.S., Lincoln C., Williams F.J., Barnes G. (1953). Bollworm and tobacco budworm as cotton pests in Louisiana and Arkansas.. La. Agric. Exp. Stn. Tech. Bull..

[42] Neunzig H.H. (1969). Biology of the Tobacco Budworm and the Corn Earworm in North Carolina.

[43] Hoffmann M.P., Wilson L.T., Zalom F.G. (1991). Area-wide pheromone trapping of *Helicoverpa zea* and *Heliothis phloxiphaga* (Lepidoptera: Noctuidae) in the Sacramento and San Joaquin valleys of California. J. Econ. Entomol..

[44] Coop L.B., Drapek R.J., Croft B.A., Fisher G.C. (1992). Relationship of corn earworm (Lepidoptera: Noctuidae) pheromone catch and silking to infestation levels in Oregon sweet corn. J. Econ. Entomol..

[45] Tobin P.C., Nagarkatti S., Loeb G., Saunders M.C. (2008). Historical and projected interactions between climate change and insect voltinism in a multivoltine species. Global Change Biol..

[46] Morey A.C., Hutchison W.D., Venette R.C., Burkness E.C. (2012). Cold hardiness of *Helicoverpa zea* (Lepidoptera: Noctuidae) pupae. Environ. Entomol..

[47] Ditman L.P., Weiland G.S., Guill J.H. (1940). The metabolism in the corn earworm. J. Econ. Entomol..

[48] Eger J.E., Witz J.A., Hartstack W., Sterling W.L. (1982). Survival of pupae of *Heliothis virescens* and *Heliothis zea* (Lepidoptera: Noctuidae) at low temperatures. Can. Entomol..

[49] Ziter C., Robinson E.A., Newman J.A. (2012). Climate change and voltinism in Californian insect pest species: Sensitivity to location, scenario and climate model choice. Glob. Chang. Biol..

[50] Masson-Delmotte V., Zhai P., Pirani A., Connors S.L., Péan C., Berger S., Caud N., Chen Y., Goldfarb L., Gomis M.I., IPCC (2021). Summary for Policymakers. Climate Change 2021: The Physical Science Basis. Contribution of Working Group I to the Sixth Assessment Report of the Intergovernmental Panel on Climate Change.

[51] Coley P.D. (1998). Possible effects of climate change on plant/herbivore interactions in moist tropical forests. Clim. Chang..

[52] Cannon R.J.C. (1998). The implications of predicted climate change for insect pests in the UK, with emphasis on non-indigenous species. Glob. Chang. Biol..

[53] IPCC Climate Change 2014: Synthesis Report (2014). Contribution of Working Groups I, II and III to the Fifth Assessment Report of the Intergovernmental Panel on Climate Change.

[54] Ockendon N., Baker D.J., Carr J.A., White E.C., Almond R.E.A., Amano T., Bertram E., Bradbury R.B., Bradley C., Butchart S.H.M. (2014). Mechanisms underpinning climatic impacts on natural populations: Altered species interactions are more important than direct effects. Glob. Chang. Biol..

[55] Parmesan C., Hanley M.E. (2015). Plants and climate change: Complexities and surprises. Ann. Bot-Lond..

[56] Stange E.E., Ayres M.P. (2010). Climate change impacts: Insects. Encyclopedia of Life Sciences (ELS).

[57] Mattson W.J., Haack R.A. (1987). Role of drought in outbreaks of plant-eating insects. Bioscience.

[58] Allen C.D., Macalady A.K., Chenchouni H., Bachelet D., McDowell N., Vennetier M., Kitzberger T., Rigling A., Breshears D.D., Hogg E.H.T. (2010). A global overview of drought and heat-induced tree mortality reveals emerging climate change risks for forests. For. Ecol. Manag..

[59] Choi W.I.L. (2011). Influence of global warming on forest coleopteran communities with special reference to ambrosia and bark beetles. J. Asia Pac. Entomol..

[60] Sallé A., Nageleisen L., Lieutier F. (2014). Bark and wood boring insects involved in oak declines in Europe: Current knowledge and future prospects in a context of climate change. For. Ecol. Manag..

[61] Stireman J.O., Dyer L.A., Janzen D.H., Singer M.S., Lill J.T., Marquis R.J., Ricklefs R.E., Gentry G.L., Hallwachs W., Coley P.D. (2005). Climatic unpredictability and parasitism of caterpillars: Implications of global warming. Proc. Natl. Acad. Sci. USA.

[62] Hance T., van Baaren J., Vernon P., Boivin G. (2007). Impact of extreme temperatures on parasitoids in a climate change perspective. Annu. Rev. Entomol..

[63] Klapwijk M.J., Ayres M.P., Battisti A., Larsson S., Barbosa P., Letourneau D.K., Agrawal A.A. (2012). Assessing the impact of climate change on groundwater quality in Turkey. Insect Outbreaks Revisited.

[64] Skelly D.K., Joseph L.N., Possingham H.P., Freidenburg L.K., Farrugia T.J., Kinnison M.T., Hendry A.P. (2007). Evolutionary responses to climate change. Conserv. Biol..

[65] Tiberi R., Branco M., Bracalini M., Croci F., Panzavolta T. (2016). Cork oak pests: A review of insect damage and management. Ann. For. Sci..

[66] Weiskopf S.R., Rubenstein M.A., Crozier L.G., Gaichas S., Griffis R., Halofsky J.E., Hyde K.J.W., Morelli T.L., Morisette J.T., Muñoz R.C. (2020). Climate change effects on biodiversity, ecosystems, ecosystem services, and natural resource management in the United States. Sci. Total Environ..

[67] Paul M., Dangol S., Kholodovsky V., Sapkota A.R., Negahban-Azar M., Lansing S. (2020). Modeling the Impacts of Climate Change on Crop Yield and Irrigation in the Monocacy River Watershed, USA. Climate.

[68] Amaducci S., Zatta A., Pelatti F., Venturi G. (2008). Influence of agronomic factors on yield and quality of hemp (*Cannabis sativa* L.) fibre and implication for an innovative production system. Field Crop. Res..

[69] Overpeck J.T., Udall B. (2020). Climate change and the aridification of North America. Proc. Natl. Acad. Sci. USA.

[70] Williams A.P., Cook E.R., Smerdon J.E., Cook B.I., Abatzoglou J.T., Bolles K., Baek S.H., Badger A.M., Livneh B. (2020). Large contribution from anthropogenic warming to an emerging North American megadrought. Science.

[71] Breshears D.D., Cobb N.S., Rich P.M., Price K.P., Allen C.D., Balice R.G., Romme W.H., Kastens J.H., Floyd M.L., Belnap J. (2005). Regional vegetation die-off in response to global-change-type drought. Proc. Natl. Acad. Sci. USA.

[72] Abatzoglou J.T., Williams A.P. (2016). Impact of anthropogenic climate change on wildfire across western US forests. Proc. Natl. Acad. Sci. USA.

[73] Citterio S., Santagostino A., Fumagalli P., Prato N., Ranalli P., Sgorbati S. (2003). Heavy metal tolerance and accumulation of Cd, Cr and Ni by *Cannabis sativa* L.. Plant Soil.

[74] Burrows W.J., Carr D.J. (1969). Effects of flooding the root system of sunflower plants on the cytokinin content in xylem sap. Physiol. Plant..

[75] Itai C., Ben-Zioni A., Bieleski R.L., Ferguson A.R., Cresswell M.M. (1974). Regulations of plant response to high temperature. Mechanisms of Regulation of Plant Growth.

[76] Itai C., Richmond A., Vaadia Y. (1968). The role of root cytokinins during water and salinity stress. Israel J. Bot..

[77] Itai C., Ben-Zioni A., Ordin L. (1973). Correlative changes in endogenous hormone levels and shoot growth induced by short heat treatments to the root. Physiol. Plant..

[78] Freeman D.C., Harper K.T., Charnov E.L. (1980). Sex change in plants: Old and new observations and new hypotheses. Oecologia.

[79] Itai C., Vaadia Y. (1965). Kinetin-like activity in root exudate of water-stressed sunflower plants. Physiol. PIant..

[80] Itai C., Vaadia Y. (1970). Cytokinin activity in water-stressed shoots. Plant Physiol..

[81] Chailakhyan M.K. (1979). Genetic and hormonal regulation of growth, flowering, and sex expression in plants. Am. J. Bot..

[82] Parker L., Abatzoglou J. (2019). Warming Winters Reduce Chill Accumulation for Peach Production in the Southeastern United States. Climate.

[83] Petersen L. (2019). Impact of Climate Change on Twenty-First Century Crop Yields in the U.S. Climate.

[84] Watson D.P., Clarke R.C. (2014). Genetic Future of Hemp. http://www.internationalhempassociation.org/jiha/jiha4111.html.

[85] Huberty A.F., Denno R.F. (2004). Plant water stress and its consequences for herbivorous insects: A new synthesis. Ecology.

[86] Netherer S., Schopf A. (2010). Potential effects of climate change on insect herbivores in European forests—General aspects and the pine processionary moth as specific example. For. Ecol. Manag..

[87] Bi J.L., Murphy J.B., Felton G.W. (1997). Antinutritive and oxidative components as mechanisms of induced resistance in cotton to *Helicoverpa zea*. J. Chem. Ecol..

[88] Bi J.L., Felton G.W., Mueller A.J. (1994). Induced resistance in soybean to *Helicoverpa zea*: Role of plant protein quality. J. Chem. Ecol..

[89] Felton G.W., Summers C.B., Mueller A.J. (1994). Oxidative responses in soybean foliage to herbivory by bean leaf beetle and three-cornered alfalfa hopper. J. Chem. Ecol..

[90] Felton G.W., Bi J.L., Mueller A.J., Duffey S.S. (1994). Potential role of lipoxygenases in defense against insect herbivory. J. Chem. Ecol..

[91] Bi J.L., Felton G.W. (1995). Foliar oxidative stress and insect herbivory: Primary compounds, secondary metabolites, and reactive oxygen species as components of induced resistance. J. Chem. Ecol..

[92] Jackson B., Gilbert L., Tolosa T., Henry S., Volkis V., Zebelo S. (2021). The impact of insect herbivory in the level of cannabinoids in CBD hemp varieties. Res. Sq..

[93] Bengtsson M., Karpati Z., Szöcs G., Reuveny H., Yang Z., Witzgall P. (2006). Flight tunnel responses of Z strain European corn borer females to corn and hemp plants. Environ. Entomol..

[94] Sudbrink D.L., Grant J.F. (1995). Wild host plants of *Helicoverpa zea* and *Heliothis virescens* (Lepidoptera: Noctuidae) in Eastern Tennessee. Environ. Entomol..

[95] Britt K.E., Reed D., Kuhar T.P. (2021). Evaluation of biological insecticides to manage corn earworm in CBD hemp, 2020. Arthropod Manag. Tests.

[96] Doughty H.B., Britt K.E., Kuhar T.P. (2020). Evaluation of biological insecticides to control corn earworm in hemp, 2019. Arthropod Manag. Tests.

[97] Britt K.E., Kuhar T.P. (2020). Laboratory bioassays of biological/organic insecticides to control corn earworm on hemp in Virginia, 2019. Arthropod Manag. Tests.

[98] White T.C.R. (1969). An index to measure weather-induced stress of trees associated with outbreaks of psyllids in Australia. Ecology.

[99] Stamp N. (2003). Out of the quagmire of plant defense hypotheses. Q. Rev. Biol..

[100] Haber A.I., Sustache J.R., Carr D.E. (2018). A generalist and a specialist herbivore are differentially affected by inbreeding and trichomes in *Mimulus guttatus*. Ecosphere.

[101] Inbar M., Doostdar H., Mayer R.T. (2001). Suitability of stressed and vigorous plants to various insect herbivores. Oikos.

